# Physical Exercise, Social Interaction, Access to Care, and Community Service: Mediators in the Relationship Between Socioeconomic Status and Health Among Older Patients With Diabetes

**DOI:** 10.3389/fpubh.2020.589742

**Published:** 2020-10-09

**Authors:** Qingwen Deng, Wenbin Liu

**Affiliations:** Department of Health Management, School of Public Health, Fujian Medical University, Fuzhou, China

**Keywords:** socioeconomic status, diabetes, health services research, quality of life, older patients, China

## Abstract

The differences in socioeconomic status (SES) will cause a disparity in the health of the elderly. Taking diabetes as an example, previous studies have focused on risk factors of diabetes, while the relationship and mechanism between SES, multi-faceted factors, and the health of older patients with diabetes are not well-understood. This study aims to investigate the association between SES and health in older patients with diabetes and the interrelated mediators between them. Based on the data of the Chinese Longitudinal Healthy Longevity Survey (CLHLS) in 2018, structural equation modeling (SEM) was used to test whether physical exercise, social interaction, access to care, and community service mediated the effect of SES on the health in older patients with diabetes. We found support for the model in which SES predicted the health in older patients with diabetes (comparative fit index = 0.910, incremental fit index = 0.911, goodness-of-fit index = 0.982, adjusted goodness-of-fit index = 0.959, standardized root mean square residual = 0.037, and root mean square error of approximation = 0.061). The total indirect effect of SES on the health accounted for 55.52% of the total effect. Results indicated that physical exercise (β = 0.108, *p* < 0.01), social interaction (β = 0.253, *p* < 0.001), and community service (β = 0.111, *p* < 0.001) had significant positive effects on the health of older patients with diabetes. SES was positively associated with physical exercise (β = 0.417, *p* < 0.001) and community service (β = 0.126, *p* < 0.01). Although no direct effect of SES on the health was found, SES mediated the positive effect in their relationship by physical exercise (indirect effect = 0.045, *p* < 0.01), and community service (indirect effect = 0.014, *p* < 0.05). This study showed the health disparities of older patients with diabetes were influenced by individual-level (physical exercise, social interaction) and environmental-level (community service). It suggests that a lack of physical exercise and health-related community service may impair the health of older patients with diabetes with low SES, which recommends individuals' positive actions and environmental supports for promoting health of regarding population.

## Introduction

Socioeconomic status (SES) is an overall measure of an individual's position in society relative to others based on a combination of education, occupation and income (1, 2). As the health disparities between the socially advantaged and disadvantaged populations become much wider (3), the impact of SES on health has increasingly been explored. A positive association between SES and health among older adults has been reported in many previous studies, which revealed that the higher the SES the better the health (4–7). In addition to SES, a set of social determinants of health, which includes health behavior (e.g., lifestyle and behavioral risk factors) and environmental factors (e.g., neighborhood and health system factors), were also reported as having impact on health status.

These findings were in line with the Anderson's model (Behavioral Model of Health Services Use) (8), which includes four important components, namely, environment, population characteristics, health behavior, and outcomes. The Anderson's model preliminarily showed the relationship between SES and other elements while influencing the health, which provided theoretical clues for further clarifying the mechanism. For instance, in Kino's study, it demonstrated that SES (high education and income predict health) predict health, and it was also confirmed that SES were associated with the adoption of health behavior and the availability of health resources (9). However, the overall mechanism by which SES affects health remains largely unknown, especially for the population with certain age-related disease.

For instance, diabetes is a common chronic and age-related disease in older adults, which has become a leading challenge of global public health due to its high incidence, disability and mortality (10). As reported by the International Diabetes Federation, China is becoming the epicenter of the diabetes epidemic with 28% of the world's older patients with diabetes live in (11). The focus of previous studies in diabetes has largely lied in the risk of the disease (12), and the relationship between the increased prevalence of diabetes and some single factors, such as low SES (13, 14), unhealthy living habits (15), poor community conditions (16), and so on, have been confirmed. However, there was still very few studies have looked at the relationship between these factors mentioned above and the health of patients with diabetes. For example, a study conducted in Korea has indicated that unfavorable socioeconomic status and adverse lifestyle behaviors negatively predicted poor health status of Korean adults with diabetes (17). And another study has found that race or ethnicity was independent predictor of health decline among older patients with diabetes in the USA (5). To be more specific, the potential mediators or other relationships formed by the interaction of SES, other multiple factors and health are not well-understood. Therefore, this study aims to investigate the association between SES and the health in older patients with diabetes and the interrelated mediators between them.

## Materials and Methods

### Theoretical Framework

**Figure 1:**
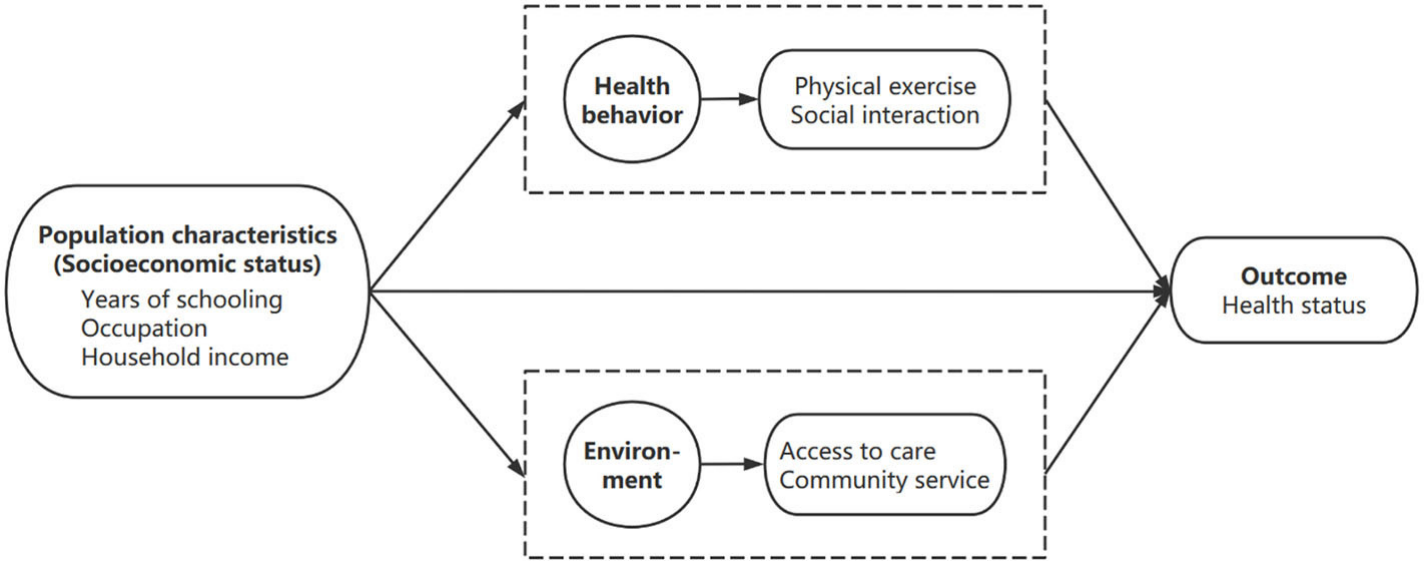
Theoretical framework.

The theoretical framework of this study was adapted from the Anderson's model. Among the four important components in the model, health behavior and environment were taken as mediating domains to investigate the relationship between population characteristics and health outcomes. Health behavior included physical exercise and social interaction, while environment included access to care and community service. Based on the above understanding, we proposed a theoretical framework (Figure 1) of this study.

### Data Resource

This study used the data of the cross-sectional survey in 2018 from the Chinese Longitudinal Healthy Longevity Surveys (CLHLS), a nationally representative and public dataset basing on a selected sample of older adults from 22 out of the 31 provinces of mainland China. All these populations represent about 85% of the total population of China (18). The CLHLS has established the sampling frame with all centenarians from the sampled counties/cities. Each sampled centenarian was matched to one octogenarian and non-agenarian that were randomly selected based on their code; for every three sampled centenarians, four older adults aged 65–79 were randomly chosen from a nearby geographical unit. The data were obtained through in-home interviews using internationally compatible questionnaire, and all investigators were trained in advance. To ensure the quality of data, the CLHLS has taken various measures in terms of proxy use, non-response rate, sample attrition, reliability and validity of major health measures, and rates of logically inconsistent answers. For example, when the interviewees are unable to answer the questions, a close family member or another proxy will provide the answers, but questions such as self-rated health, life satisfaction, and cognitive tests are answered by the interviewees only. The surveyed individuals over 65 years old with diabetes diagnosed by a physician will be included in this study, except for the samples with missing values in any variables of interest as mentioned below.

### Measurements

#### Outcome Variable

The Quality of Well-being Scale (QWB) developed by Kaplan and Anderson (19) was used as the outcome variable in this study. QWB is a common indicator to measure health that reflects both the objective indicators and subjective evaluation of personal health status (20), which combines preference-weighted measures of symptoms and functions. The QWB is ranging from 0 (for death) to 1.0 (for asymptomatic full function). Table 1 describes the items contained in QWB, the weight of relevant items, calculation formula (19), and corresponding variables in the CLHLS data.

**Table 1:** QWB's items, weights, calculation formula, and corresponding variables in the CLHLS data.

**Step No**.	**Definition**	**Corresponding variables in the CLHLS data**	**Weight**
	**Mobility Scale (MOB)**	e14, g131, g132	
5	No limitations for health reasons		0.000
4	Did not drive a car, health related; did not ride in a car as usual for age (younger than 15 year), health related, and/or did not use public transportation, health related; or had or would have used more help than usual for age to use public transportation, health related		−0.062
2	In hospital, health related		−0.090
	**Physical Activity Scale (PAC)**	e4, e11~e13, g9, g131	
4	No limitations for health reasons		0.000
3	In wheelchair, moved or controlled movement of wheelchair without help from someone else; or had trouble or did not try to lift, stoop, bend over, or use stairs or inclines, health related; and/or limped, used a cane, crutches, or walker, health related; and/or had any other physical limitation in walking, or did not try to walk as far or as fast as others the same age are able, health related		−0.060
1	In wheelchair, did not move or control the movement of wheelchair without help from someone else, or in bed, chair, or couch for most or all of the day, health related		−0.077
	**Social Activity Scale (SAC)**	e0~e10	
5	No limitations for health reasons		0.000
4	Limited in other (e.g., recreational) role activity, health related		−0.061
3	Limited in major (primary) role activity, health related		−0.061
2	Performed no major role activity, health related, but did perform, self-care activities		−0.061
1	Performed no major role activity, health related, and did not perform or had more help than usual in performance of one or more self-care activities, health related		−0.106
	**Symptom/Problem Complexes (CPX)**	b34, b36, b38, e4, g106, g131, g15e~h1, g15j~k1, g15m~q1, g15a~y3, g22, g24, g181	
	There are 23 categories in total. Detailed indicators and weights can be found in Table 2 in Kaplan and Anderson (19)		

Formula: W = 1 + (CPXωt) + (MOBωt) + (PACωt) + (SACωt).

*where ωt is the preference-weighted measure for each indicator. For example, a person's MOB, PAC, SAC, and CPX, respectively corresponds to 4, 3, 3, and 11, the W score for he/she is W = 1 + (−0.257) + (−0.062) + (−0.060) + (−0.061) = 0.56*.

#### Explanatory Variable

SES was the explanatory variable of this study, which was measured by asking three questions: “years of schooling,” “main occupation before age 60,” and “total income of your household last year.” Years of schooling was classified into three categories: 0 years (1), 1~5 years (2), and 6 years or more (3), respectively referred to uneducated, primary school, and middle school or more. The categories of occupation in the questionnaire included professional and technical personnel, governmental, institutional or managerial personnel, commercial, service or industrial worker, self-employed, agriculture, forestry, animal husbandry or fishery worker, house worker, and others. In this study, occupation was recoded into two categories according to occupational characteristics: manual worker (1), including commercial or industrial worker, farmer, self-employed, house worker, and others; and non-manual worker (2), including professional, technical or managerial personnel; Household income was divided into four quartiles with quintile 1 (1) indicating the poorest and quintile 4 (4) indicating the richest.

#### Mediating Variables

To evaluate the pathways through which SES affected the health of older patients with diabetes, physical exercise and social interaction at the individual level, as well as access to care and community service at the environmental level, were all considered as potential mediators. The question “Do you regularly exercise in the past, such as playing ball, running and Qigong?” was used to collect information on physical exercise, and the responses to this question were dichotomized into no (1) or yes (2). Given the social background and feature of times for the social interaction of Chinese older adults, the measurement of social interaction included three indicators: the frequencies of participation in group leisure activities (i.e., square dancing, playing cards/mah-jongg), informal interaction (series, interact with friends), and organized social activities. A score was given to each indicator based on five responses: almost every day (5), not daily, but once for a week (4), not weekly, but at least once for a month (3), not monthly, but sometimes (2), and never (1). And the highest frequency of the three kinds of indicators was deemed as the frequency of an individual's social interaction. Access to care was assessed by one question “Can you get medical service in time?” Responses to this question were dichotomized into no (1) or yes (2). Community service was measured with two indicators: the availability of healthcare (i.e., home visit services, healthcare education) and psychological comfort services (i.e., psychological consulting services, social and recreation services) in your community. Options for both questions include no (1) and yes (2). The score for community service was recoded after merging options of the two questions as four classes: both (4), the former (3), the latter (2), and neither (1).

#### Covariates

The respondents' sex and age were the covariates in this study. Females and males were coded as 1 and 2. Ages were classified into three groups: 65~74 years old (3), 75~89 years old (2), 90 years old and above (1).

### Statistical Analysis

Frequency and percentage were used to describe sample characteristics. Structural equation modeling (SEM) method was applied to test the relationship between SES and health condition of older patients with diabetes as well as the mediating effect of physical exercise, social interaction, access to care, and community service. Following fit indices were used to evaluate the model fit: comparative fit index (CFI), incremental fit index (IFI), the goodness-of-fit index (GFI), the adjusted goodness-of-fit index (AGFI), standardized root mean square residual (SRMR), and the root mean square error of approximation (RMSEA). We aimed at an adequate fit: CFI ≥ 0.90, IFI ≥ 0.90, GFI ≥ 0.90, AGFI ≥ 0.90, SRMR ≤ 0.08, RMSEA ≤ 0.08. The parameters were estimated by the maximum likelihood method. All the analyses were performed with M*plus* 8.0 software.

## Results

Of the 15,874 individuals aged 65 and over based on the 2018 CLHLS survey, 1,423 had diabetes. After excluding the sample with missing values in any variables of interest, a total of 1,030 respondents (older patients with diabetes) were included in the final analyses.

Table 2 shows the individual characteristics and the QWB scores of these 1,030 respondents. The respondents comprised of 57.1% (*n* = 588) females, 81.9% (*n* = 844) were under 90 years old, 57.0% (*n* = 587) were married and lived with their spouse, and 72.5% (*n* = 747) lived in the urban area. Among the respondents, 45.0% (*n* = 463) had more than 6 years of schooling, 78.5% (*n* = 809) mainly engaged in manual labor before 60 years old, and 31.3% (*n* = 322) were in the highest quartile of household income, i.e., the total household income of more than 100,000 yuan. The prevalence of past exercising was estimated to be 43.8% (*n* = 451). Of social interaction, 32.8% (*n* = 338) participated almost daily, whereas 30.9% (*n* = 318) never participated. Nearly all the respondents (98.3%, *n* = 1013) reported that they could get adequate medical service in time. About one third (33.0%, *n* = 340) of the respondents had both healthcare and psychological comfort services available in the community, while another one third (30.7%, *n* = 316) had neither of these two services. The average value of the QWB scores was 0.6.

**Table 2:** Characteristics of the sample (*N* = 1030).

**Characteristic**	***N* or mean**	**% or SD**
**Sex**		
female	588	57.1
male	442	42.9
**Age (years old)**		
90 and above	186	18.1
75~89	502	48.7
65~74	342	33.2
**Marital status**		
Other	443	43.0
Married and living with spouse	587	57.0
**Residential area**		
Rural	283	27.5
Urban	747	72.5
**Years of schooling (years)**		
0	333	32.3
1~5	234	22.7
≥6	463	45.0
**Occupation**		
Manual worker	809	78.5
Non-manual worker	221	21.5
**Household income**		
Q1	221	21.5
Q2	248	24.1
Q3	239	23.2
Q4	322	31.3
**Regularly exercised in the past**		
No	579	56.2
Yes	451	43.8
**Frequency of social interaction**		
Never	318	30.9
Not monthly, but sometimes	95	9.2
At least once for a month	87	8.4
Once for a week	192	18.6
Almost everyday	338	32.8
**Can you get medical service in time**		
No	17	1.7
Yes	1013	98.3
**Are healthcare or psychological comfort services available in your community**		
Neither	316	30.7
The former	320	31.1
The latter	54	5.2
Both	340	33.0
**QWB**	0.6	0.1

In order to test the proposed framework, we constructed several models. Model fit indices for all models are summarized in Table 3. Model 0 was the base model that no mediations involved, which showed poor model fit. Model 1 posited physical exercise as a mediator of the effect of SES on the health of older patients with diabetes, which still fitted poorly. Model 2 added social interaction as the second mediator to Model 1. Model 3 set physical exercise, social interaction, and community service as mediators. Model 4 allowed the effect of SES to be mediated via variables of Model 3 plus additional access to care. The fitting statistics of Model 2 to Model 4 met the criteria, among which, model 4 incorporated all the mediator hypotheses and generated the best fit statistics.

**Table 3:** Model fit indices of potential models.

**Model**	**Fit indices**							
	***χ*****^2^**	**df**	**GFI**	**AGFI**	**CFI**	**IFI**	**SRMR**	**RMSEA**
Model 0	22.370	2	0.978	0.889	0.884	0.885	0.044	0.144
Model 1	48.083	4	0.981	0.930	0.911	0.912	0.038	0.103
Model 2	58.991	7	0.981	0.943	0.920	0.921	0.041	0.085
Model 3	74.697	11	0.980	0.949	0.907	0.908	0.040	0.075
Model 4	77.537	16	0.982	0.959	0.910	0.911	0.037	0.061

**Figure 2:**
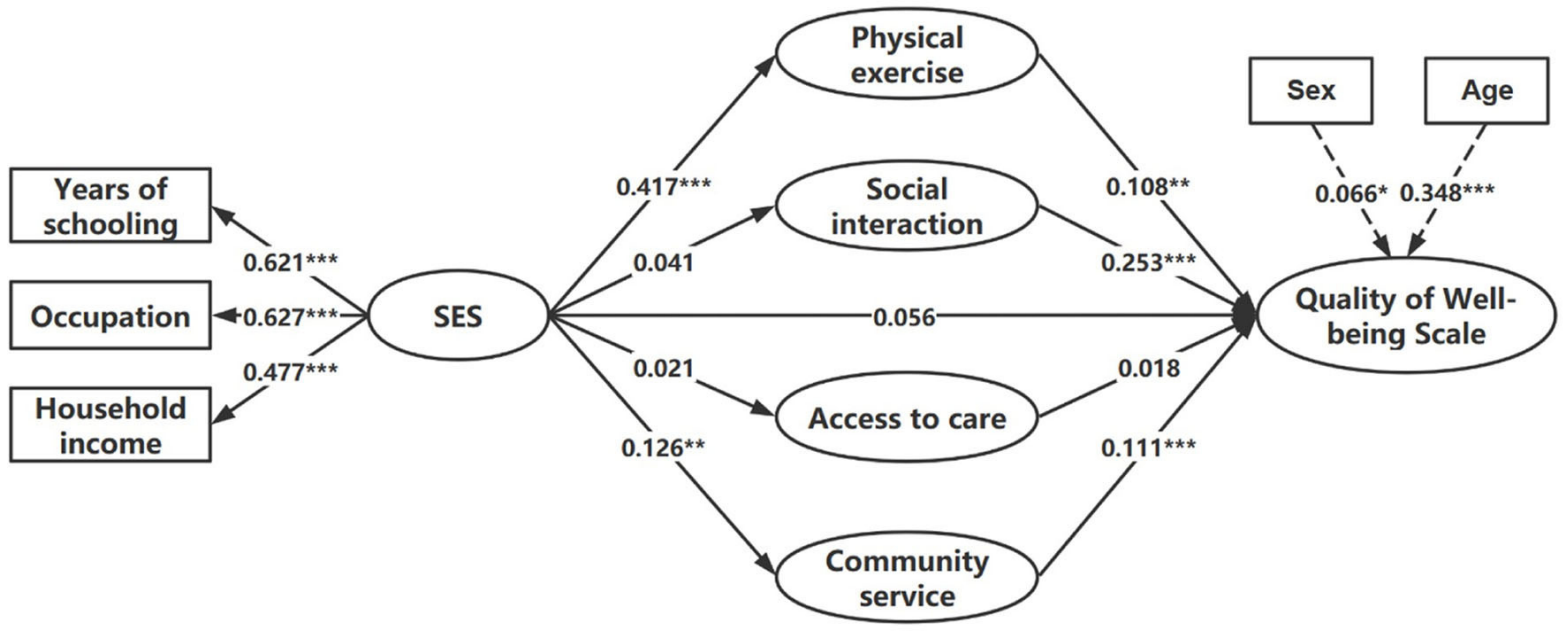
Final model of the association between SES and the QWB of older patients with diabetes (*p < 0.05; **p < 0.01; ***p < 0.001).

Model 4, namely the final model, was illustrated in Figure 2. Table 4 displays a decomposition of the direct and total effect of SES on the QWB among older patients with diabetes, the specific indirect effect through four mediations, and the ratio of indirect effect to total indirect effect. In the final Model, after controlling for respondents' sex and age, the total effect of SES on the QWB of older patients with diabetes was 0.125 (*p* < 0.01). SES positively predicted physical exercise (β = 0.417, *p* < 0.001) and community service (β = 0.126, *p* < 0.01). Social interaction (β = 0.253, *p* < 0.001) had the largest direct effect on the QWB of older patients with diabetes, followed by community service (β = 0.111, *p* < 0.001), and physical exercise (β = 0.108, *p* < 0.01). Additionally, although the model did not show a direct effect of SES on the QWB for older adults with diabetes (*p* > 0.05), SES mediated the effect in their relationship through physical exercise (indirect effect = 0.045, *p* < 0.01) and community service (indirect effect = 0.014, *p* < 0.05).

**Table 4:** Mediators in the association between SES and the QWB of older patients with diabetes.

**Variable**	**Model 0**	**Model 1**	**Model 2**	**Model 3**	**Model 4**
**Socioeconomic status**					
Direct effect on the QWB	0.111^**^	0.059	0.075	0.056	0.056
Total effect	0.111^**^	0.112^**^	0.127^**^	0.125^**^	0.125^**^
**Physical exercise**					
Direct effect on the QWB	-	0.126^***^	0.100^**^	0.108^**^	0.108^**^
Indirect effect (SES × physical exercise)	-	0.053^**^	0.042^**^	0.045^**^	0.045^**^
The ratio of indirect effect to total indirect effect (%)	-	1	80.77	65.22	65.22
**Social interaction**					
Direct effect on the QWB	-	−	0.258^***^	0.253^**^	0.253^***^
Indirect effect (SES × social interaction)	-	−	0.010	0.010	0.010
The ratio of indirect effect to total indirect effect (%)	-	−	19.23	14.49	14.49
**Community service**					
Direct effect on the QWB	-	−	−	0.111^***^	0.111^***^
Indirect effect (SES × community service)	-	−	−	0.014^*^	0.014^*^
The ratio of indirect effect to total indirect effect (%)	-	−	−	20.29	20.29
**Access to care**					
Direct effect on the QWB	-	−	−	−	0.018
Indirect effect (SES × access to care)	-	−	−	−	0.000
The ratio of indirect effect to total indirect effect (%)	-	−	−	−	0.00

* p < 0.05,

** p < 0.01,

*** *p < 0.001*.

## Discussion

To the best of our knowledge, there was a dearth of studies on the relationship between SES and health among older patients with diabetes. This study not only examined this relationship, but also determined whether SES mediated the health of older patients with diabetes through four latent variables: physical exercise, social interaction, access to care, and community service. Although the direct influence of SES on the health status of older patients with diabetes was not found in the final model, mediation roles of physical exercise and community service in their relationship were observed in this study. Additionally, physical exercise, social interaction, and community service showed significant effects on the health of older patients with diabetes.

At the individual level, the findings showed that the SES of older patients with diabetes positively predicted their physical exercise, while its impact on social interaction was not significant. Meanwhile, both physical exercise and social interaction have a positive effect on the health of older patients with diabetes. As a result, SES mediated health through physical exercise rather than social interaction. On the one hand, higher SES tended to have a better sense of control over their life outcomes (21), which associated with good adherence to health behavior and regular physical exercises (22). And the regular physical exercise has been shown to be an outstanding way to improve physical and mental health (23–25), for example, controlling blood glucose, preventing and treating depression and reducing the risk of cardiovascular disease (26). These would explain the significant association between SES and physical exercise, as well as between physical exercise and health of older patients with diabetes. On the other hand, among the three categories of social interaction, namely group leisure activities, informal interaction and organized social activities, participating in group leisure activities (square dancing and playing mah-jongg/cards are the most common forms) and informal social interaction among relatives and friends were the most leading aspects of social relationships for the elderly in China, regardless of social class (27). This may explain why SES did not have a significant impact on social interaction. However, the positive effects of social interaction on health have been confirmed by many studies (28, 29). Psychologically, social interaction can help older patients with diabetes find emotional support, enhance self-efficacy and reduce psychological problems, as older patients with diabetes report more depression (30, 31). Physically, social interaction benefits the health of older patients with diabetes by keeping them physically active. Thus, significant relationship was found between social interaction and health, rather than between SES and social interaction.

From the environmental perspective, the results indicated that richer community service was predicted by higher SES. Community service significantly affected the health of older patients with diabetes, while access to care did not. SES affected the health with mediation through community service rather than access to care. Benefiting from the health reform initiated in 2009, the coverage of primary care service institutions has been greatly expanded (32). According to the respondents included in this study, almost all of them (98.3%) were able to reach the nearest health facility in time when needed, which may result in the failure to detect significance. With respect to the relationship among the SES, community service and the health of older patients with diabetes, it may be explained that people with higher SES have higher health awareness and tend to actively seek and use relevant health services, which was also demonstrated in previous research that people with higher SES were more likely to be aware of their diabetes status and to take measures to keep healthy (13). With a wider social network and information resource, as well as better economic affordability, such people will have greater access to various services resources in the community, which would benefit disease control and health maintenance.

It is also noteworthy that no significant impact of SES was shown on the health of older patients with diabetes in this study, which was inconsistent with the results of Nicklett (5) and Lee (17) studies that low SES leads to poor health among patients with diabetes. One plausible reason may be national differences. Both Nicklett (5) and Lee (17) studies were conducted in developed countries, where negative association between SES and prevalence of diabetes have been confirmed (33). People with low SES tended to be associated with chronic stress and negative life events. They also had less access to resources and were more vulnerable to behavioral risks, which would affect health status in the long term (34). However, for the developing countries, there may be some non-negligible differences in change of behavioral lifestyle between lower and higher SES. Taking China for example, during the past four decades of rapid economic growth from a state of poverty and backwardness, the consumption of high energy diets occurs broader and faster in the lower SES than in the higher SES (13), while higher SES generally increases the adoption of sedentary habits, excessive calorie intake (35). In other words, some behavioral lifestyle risk of diabetes generally increased both in the lower and higher SES. Since there were not merely positive or negative effects, the impact of SES on the health of older patients with diabetes was not significant.

Some limitations of this study should be recognized. First, all of the data in this study were obtained from self-reported, which may result in information bias. Second, considering the limitation of a cross-sectional study in causal inferences, it may be more prudent to investigate the causality by panel session data or so on in future research. Third, since the possibility cannot be ruled out that some potential mediators between SES and the health of older patients with diabetes not included in this study, more comprehensive models should be studied in the future.

## Conclusion

In this study, we evaluated the relationship between SES and the health of older patients with diabetes, as well as the mediating roles of physical exercise, social interaction, access to care, and community service. The findings showed SES probably enhanced health by increasing regular exercise and providing more community service, which indicated that health-related individual behaviors and environmental supports can mediate the relationship between SES and the health of older patients with diabetes, and relieve the health disadvantages cumulated by SES in old age. To improve the health of older patients with diabetes and create healthier aging, it requires not only the individuals' initiatives and positive actions, such as keeping physical and mental health through exercising and socializing, but also the support of the environment, such as making health-related resources and services available in the community and residence.

## Data Availability Statement

Publicly available datasets were analyzed in this study. This data can be found here: http://162.105.138.117/dataset.xhtml?persistentId=doi:10.18170/DVN/WBO7LK.

## Ethics Statement

The studies involving human participants were reviewed and approved by the Research Ethics Committees of Peking University. The patients/participants provided their written informed consent to participate in this study.

## Author Contributions

WL and QD contributed to the conception and design of the study. QD conducted the data reduction, analyses, and wrote the manuscript. WL guided the whole process and reviewed the manuscript. All authors read and approved the manuscript before submission.

## Conflict of Interest

The authors declare that the research was conducted in the absence of any commercial or financial relationships that could be construed as a potential conflict of interest.
